# Immunization Offer Targeting Migrants: Policies and Practices in Italy

**DOI:** 10.3390/ijerph15050968

**Published:** 2018-05-12

**Authors:** Teresa Dalla Zuanna, Martina Del Manso, Cristina Giambi, Flavia Riccardo, Antonino Bella, Maria Grazia Caporali, Maria Grazia Dente, Silvia Declich, The Italian Survey CARE Working Group

**Affiliations:** 1 Department of Cardiac, Thoracic and Vascular Sciences, and Public Health, University of Padova, 35122 Padova , Italy; teresadallazuanna@gmail.com; 2 National Institute of Health (Istituto Superiore di Sanità, ISS), viale Regina Elena, 299-00161 Rome, Italy; martina.delmanso@iss.it (M.D.M.); cristina.giambi@iss.it (C.G.); flavia.riccardo@iss.it (F.R.); antonino.bella@iss.it (A.B.); mariagrazia.caporali@iss.it (M.G.C.); mariagrazia.dente@iss.it (M.G.D.)

**Keywords:** refugee health, asylum seekers, migrants, infectious diseases, vaccination, Italy

## Abstract

The unprecedented flow of migrants over the last three years places Italy in front of new issues regarding medical care from the rescue phase up to the integration into the national health services, including preventive actions. We used online questionnaires to investigate the Italian national and regional policies for immunization offer targeting asylum seekers, refugees, irregular migrants and unaccompanied minors. Another questionnaire was used to assess how these policies are translated into practice in migrant reception centres and community health services. Questionnaires were filled out at the national level, in 14 out of 21 Regions/Autonomous Provinces, and in 36 community health services and 28 migrant reception centres. Almost all responders stated that all vaccinations included in the National Immunization Plan are offered to migrant children and adolescents. The situation concerning adults is fragmented, with most of the Regions and local centres offering more vaccines than the national offer—which include polio, tetanus and measles–mumps-rubella. Data on immunized immigrants is archived at the regional/local level with different methods and not available at the national level. Further efforts to ensure consistency in vaccine provision and adequate mechanisms of exchanging data are needed to guarantee a complete vaccination offer and avoid unnecessary health actions, including unnecessary re-vaccination.

## 1. Introduction

During 2016, 511,371 people have travelled to Europe from Africa and Asia [1]. Compared with previous years, the proportion of migrants that arrived in Italy increased due to the closure of the Balkan route in March 2016. By the end of the year, 181,436 people had crossed the Mediterranean reaching the Italian coast, approximately 30,000 more than in 2015 [2].

In Italy, all incoming migrants were hosted upon arrival in hotspots located near borders, where they were expected to stay 24 to 48 h. Here, migrants would receive a medical consultation and emergency care if in need. Migrants who did not ask for asylum were then relocated in secure governmental Centres for Identification and Expulsion (CIE), waiting to be accompanied back to their country of origin [3]. Conversely, when migrants wished to seek asylum in Italy, they were sent to the first reception centres, also known as “hubs”, non-secure large centres for asylum seekers, located throughout the Italian territory. Here, they were expected to stay for a maximum of 30 days while their asylum claim was being placed [4].

After leaving hubs, migrants seeking asylum or recognized as refugees were taken under the responsibility of a specific system designed for the protection of asylum seekers and refugees (SPRAR system). The SPRAR system offers a set of accommodation and integration services aimed at guaranteeing the protection of asylum seekers and refugees and at facilitating integrated reception at the community level [5]. The increasing number of migrants coming to Italy in the last years has made this system’s reception capacity insufficient. For this reason, since 2015, additional highly diverse accommodation structures have been authorized. These structures have been called “extraordinary holding centres” (CAS). Their aim was to reduce delays in the relocation of migrants from hubs to the SPRAR system. The migratory pressure, however, has not decreased and in 2016 there were about 3100 CAS structures that hosted more than 70% of all asylum seekers, while only 7% were in hubs, and less than 20% were hosted through the SPRAR system [6].

Migrants may experience a number of health issues caused by the living conditions faced during the migratory journey and once in migrant reception centres; overcrowding could also favour the occurrence of outbreaks. For vaccine preventable diseases (VPD), these could be fuelled by low immunization coverage among the hosted populations [7,8,9,10,11]. In November 2015, the Joint Statement of the World Health Organization (WHO), the United Nations High Commissioner for Refugees (UNHCR) and the United Nations Children’s Fund (UNICEF) [12] and the technical document issued by the European Centre for Disease, Prevention and Control (ECDC) [13] supported the implementation of a comprehensive vaccination offer targeting newly arrived migrants and stated some principles to guide this process.

Migrants residing in the Italian territory have the right to access the same community health services that are available for Italian citizens, while irregular migrants are provided with a temporary health code (STP code) to access, free of charge, ‘urgent’ or ‘essential’ care, including some preventive care [14]. The Italian National Health System (NHS) universally offers vaccinations largely free of charge [15]. According to National Immunization Plan (NIP) 2012–2014, in force in 2016, in the first 15 months of age, all children should receive the complete vaccination courses for diphtheria, tetanus, pertussis (DTP), poliomyelitis (IPV), measles-mumps-rubella (MMR), hepatitis B (HBV), haemophilus influentiae type b (HiB), pneumococcus (PCV), meningococcus C (MenC). From 6 to 15 years, children should receive booster doses for DTP, IPV, MMR, a dose of MenC if not immunized before, varicella if susceptible and at 12 years the vaccine against papilloma virus (HPV) for girls only. Adults should receive a booster dose for DTP each 10 years, MMR if susceptible and vaccination against influenza from 65 years. Additional vaccinations are provided depending on risk conditions and epidemiological situations [16].

The responsibility for the provision of health services in Italy has been gradually decentralized at sub-national level to 19 Regions and two Autonomous Provinces (AA.PP.) [17,18]. In each Region/A.P., geographically based local health units (LHU) directly deliver community health services and primary care, including vaccines in public immunization services [15]. In some cases, they are administered by paediatricians/general practitioners (GPs). Due to decentralization, some variations in the vaccination offered across the Regions/AA.PP. are possible both for Italian citizens and for migrants.

Previous studies have assessed immunization policies targeting asylum seekers, refugees, irregular migrants and unaccompanied minors among different Mediterranean countries [19,20] and few studies within specific local settings in Italy [21,22,23], but, to the best of our knowledge, nobody has explored policies and practices within the Italian territory.

The aim of this paper is to describe national and regional immunization policies targeting asylum seekers, refugees and irregular migrants in Italy and their local application in migrant reception centres and community health services.

## 2. Materials and Methods

This cross-sectional survey was part of a wider study conducted in six European countries, in the frame of the CARE project (Common Approach for Refugees’ and migrants’ health) [24].

In Italy, the survey was conducted with two questionnaires to collect data on immunization policies targeting asylum seekers, refugees, irregular migrants and unaccompanied minors at the national and regional level. A third questionnaire, to explore how national policies are applied at the local level, was developed in two versions: one for migrant reception centres and one for community health services. The questionnaires were tested by CARE project partners and modified accordingly.

The national and regional questionnaires were addressed to public health experts working in the field of infectious disease control and vaccination programmes, at the national or regional level, respectively. At the local level, we contacted migrant reception centres from a list provided by the Ministry of Interior and community health services in each Region/A.P. as suggested by the regional public health experts. For each centre/service, the request to fill in the questionnaire was addressed to either the person in charge of the centre/service or a health professional. Only centres with health professionals were asked to complete the survey and were included in the analysis.

The electronic questionnaires were developed using an online software, Survey Monkey [25], and a link to the online questionnaire was sent via email to the identified contact persons. Questions were closed-ended, with optional space for input of free text, and covered the following aspects:

i. legal framework/regulations supporting vaccination offer to asylum seekers, refugees, irregular migrants and unaccompanied minors;
ii. target groups for vaccination, assessment of immunization status and vaccination offered to migrants (children, adolescents and adults);
iii. place for vaccination delivery, availability of Standard Operating Procedures (SOPs) for migrants’ immunization;
iv. recording and transmission of data on administered vaccines, and challenges.

For migration-related definitions, we refer to the International Organization for Migration (IOM) glossary of terms [26]. The legal framework/regulation is defined as a law, a recommendation or an immunization plan supporting migrants’ vaccination. SOPs are procedures that are shaped from the legal framework and involve actions to achieve the goals set out in the strategy and imply an organization setting out the rules and monitoring their implementation [20].

The national survey was launched in October 2016, the regional and local surveys in November 2016. Data collection was completed in February 2017.

We carried out a descriptive analysis of the data collected at the national, regional and local level in Italy. We performed frequency analysis for all the categorical variables collected and the proportions of responses were summarized.

## 3. Results

### 3.1. Profile of the Responders

**Figure 1:**
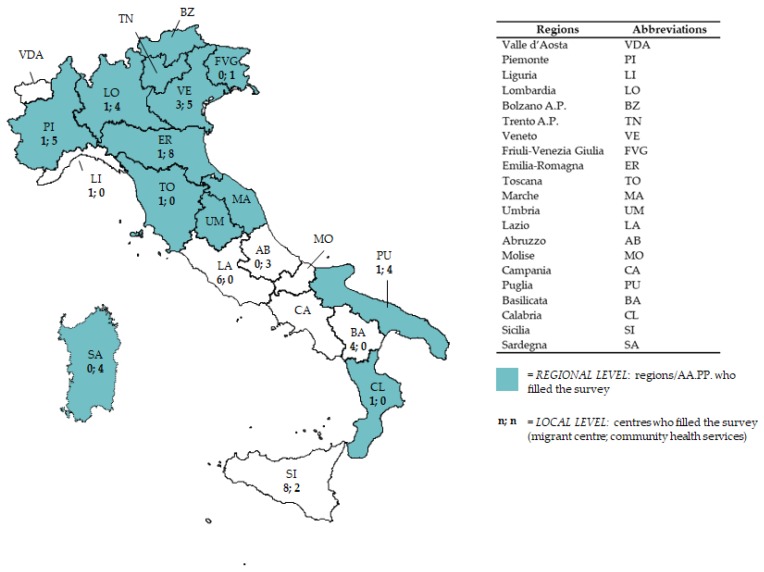
Regions/Autonomous Provinces and local centres/services responding to the questionnaire. Italy, 2017.

The national questionnaire was filled in by people in charge of the Infectious disease/VPD Unit at the Ministry of Health (MoH). We received the regional questionnaire from 14 out of 21 Regions/AA.PP.; it was filled in by the public health experts in charge of infectious diseases control at the regional level. At the local level, we received 64 questionnaires: 36 from community health services and 28 from migrant reception centres (Figure 1).

### 3.2. Vaccination Offer at National and Regional Levels

#### 3.2.1. Legal Framework Supporting Vaccination Offers Targeting Asylum Seekers, Refugees, Irregular Migrants and Unaccompanied Minors

A national legal framework has been specifically established for asylum seekers, refugees, and irregular migrants’ immunization, with several ministerial decrees since 1993 [27,28,29,30]. In addition, the National Plan for Elimination of Measles and congenital rubella 2010–2015 provided indications to increase coverage among “hard-to-reach” groups such as nomads and immigrants [31].

Eight Regions (Piemonte (PI), Bolzano A.P. (BZ), Veneto (VE), Friuli Venezia Giulia (FVG), Emilia Romagna (ER), Umbria (UM), Puglia (PU), Sicilia (SI)) reported having a regional regulation in place supporting migrants’ immunization, while the other six (Lombardia (LO), Trento A.P. (TN), Toscana (TO), Marche (MA), Calabria (CL), Sardegna (SA)) just follow the national policy (Table 1).

#### 3.2.2. Vaccination Offer to Migrant Children and Adolescents

Both at national and regional levels, responders answered that vaccinations were offered to all migrant children/adolescents regardless of their legal status (asylum seekers, refugees, irregular migrants and unaccompanied children) and without setting any limit for risk condition other than those provided in the NIP. The upper age limit for vaccination offer targeting children/adolescent migrants was 15 years for Italy and VE, BZ, UM, CL, SI and SA, or 18 years for the remaining regions.

The responder at the national level and all Regions indicated that immunization status was checked though anamnesis or verification of the immunization cards. At the national level, the responder stated that laboratory tests of immunity for tetanus and HBV is planned, if the migrants have no immunization card. Most regions also used laboratory tests mainly for tetanus and HBV.

At the national level and 13/14 Regions, migrant children and adolescents susceptible or with undocumented status were offered all the vaccinations included in the NIP appropriate for age. TN offered to migrants only vaccinations against polio, tetanus, diphtheria and MMR (Table 1).

#### 3.2.3. Vaccination Offer to Migrant Adults

At the national level, vaccinations were offered to adult migrants, regardless of their legal status and without age specification. As for ministerial decrees, particular attention was paid to migrants coming from polio endemic countries or from countries at risk of polio reintroduction, and to those with wounds at risk for tetanus infection. All 14 of the responding Regions offered immunization to adult migrants without any limit for age groups, although with differences across Regions. In addition, 13/14 Region/AA.PP. did not set any limit for any risk condition other than those provided in the NIP.

Also for adults, at national and regional levels, the immunization status was assessed through anamnesis or by verifying the immunization card. When the vaccination status was undocumented, laboratory testing was recommended at national level and performed in four Regions, mainly for tetanus and HBV (Table 1).

According to national policies, adult migrants susceptible or with an undocumented status were offered vaccinations against polio, tetanus (limited to people presenting risk conditions like exposed wounds) and MMR. Five regions reported that migrant adults received all the vaccinations included in the NIP appropriate for age, while the others indicated that only some vaccinations were offered to migrant adults (Table 2).

#### 3.2.4. Vaccine Delivery

According to national policies, vaccinations should be delivered at holding level and community level for children and adults. Vaccines were routinely administered at community level in all Regions, for all age groups (vaccination centres or primary health care centres). In six regions (TN, VE, FVG, TO, CL, SI), vaccinations are delivered also at holding level (Table 1).

Informed consent was always required: verbally in five Regions and in written form in six Regions, among the 11 Regions/P.A. (Table 1).

The responder at the national level did not indicate the presence of SOPs for vaccine delivery for migrants, while seven Regions reported to have SOPs, available in the place of vaccination delivery (BZ, TN, VE, ER, TO, UM, PU). In some Regions, SOPs contained procedures for the facilitation of access to vaccination services for children/adolescents (BZ, VE, ER, TO, UM, PU) and for adults (VE, TO, UM, PU).

#### 3.2.5. Recording of Information on Administered Vaccines and Practical Challenges

The national responder stated that there was not yet a national immunization electronic registry recording all vaccines administered to local population, nor to migrants. In addition, 10/14 Regions affirmed that data on immunised migrants were recorded in regional immunization registries, mostly in the same electronic (PI, LO, BZ, VE, FVG, ER, UM, PU) or paper based (CL, SA) registry for general population. TN and TO developed an electronic registry specifically for migrants. MA recorded the information only in individual immunization cards. In three Regions (BZ, VE, PI), methods for data recording varied by migration centre. Eight Regions made this information available: to Local Health Authorities (BZ, VE, TO, UM, PU), Regional Health Authorities-RHA (VE, FVG, ER, UM, PU), other centres where migrants were relocated (ER, TO) and to regional epidemiology centres (PI, PU).

The main practical challenges faced an immunization offer to migrants are listed in Table 3.

In addition, most Regions enlightened the difficulty of getting information on the immunization status from immunization cards because these are rarely available.

### 3.3. Vaccination Offer at Local Level

#### 3.3.1. General Information on Responding Centres

The 28 migrant reception centres that filled in the survey were 18 CAS, four centres for unaccompanied minors, five Hubs (with a capacity of 1246, 1200, 744, 496, 414 people) and one CIE (with a capacity of 219 people). Information was collected from 36 community health services from nine Regions. These centres were vaccination services (27), PHC centres (3), or centres of public health/prevention services (6). Characteristics of responding migrant and health services are summarized in Table 4.

#### 3.3.2. Immunization Practices in Migrant Reception Centres

Of the 15 migrant centres hosting minors (including 5 Hubs), 10 indicated that there was a check of immunization status, for all vaccinations included in the NIP appropriate for age. Only five of these centres (including four Hubs) indicated that they offered vaccinations to children/adolescents, all of them providing all the vaccinations included in the NIP appropriate for age. Vaccines were available in two hub centres, while, in the other three centres, they were provided by the community health services and administered by internal health staff. Informed consent is always required (written or verbal) to migrants or to their parents.

Of the 25 centres accommodating adults, seven checked the immunization status and three of them offered vaccinations to migrant adults (three Hubs, also offering vaccination to minors). One administered all vaccinations according to the NIP to adults of all ages, another addressed the vaccination offer to groups at risk only, the third administered only influenza vaccine (Table 2). Vaccines were delivered by the staff of the institution who oversaw the centre in two cases, and by staff of the vaccination service in the third case, and written informed consent was required.

Six out of 10 centres not offering vaccines to children, and 11/22 not offering vaccines to adults reported that they informed migrants on their immunization needs. In addition, sometimes dedicated health staff facilitated migrants’ access to vaccination services (in four centres for minors and five for adults), or the staff of the centre contacted the vaccination service to fix a date for the administration of vaccines (two cases), or informed the service through a systematic information flow on the number of migrants in the centre that needed to be vaccinated (three cases).

Six centres also highlighted the presence of formal agreements between the centre and the vaccination service for the provision of vaccines.

All five migrant centres that offered vaccines registered data on vaccine administration, and made them available for other institutions (Table 5). Practical challenges faced by migrant centres are listed in Table 3.

#### 3.3.3. Immunization Practices in Community Health Services

At the community level, in all 35 health services dealing with paediatric patients (the same offering vaccinations to native population), the immunization status was checked through anamnesis and vaccination card verification. Twenty-five centres reported also performing laboratory tests in case of unknown immunization status (22 for tetanus, 21 for HBV, six for MMR, six for diphtheria, five for varicella, one for the serogroups A, C, W and Y of Meningococcus and pertussis). All 35 centres also offered vaccinations to migrant children/adolescents. Thirty-three offered all vaccinations included in the NIP appropriate for age, one limited the offer to polio, diphtheria, tetanus, HBV, influenza, and MenC, the other to polio, DTP, HBV, Hib, PCV, and MMR. Informed consent was required in 34 out of 35 centres, written in 23 of them.

Twenty-six of the 32 health services dealing with adults reported verifying the immunization status of migrant adults, through anamnesis or by viewing the vaccination card. Twelve centres also provided laboratory testing, if necessary, for HBV (11), tetanus (5), MMR (3), and HAV (1). Thirty out of 32 centres indicated that they offered vaccinations to migrant adults, most of them without any limit related to age or to specific conditions (one specified that the offer was limited to adults from Afghanistan, Cameroon, Equatorial Guinea, Ethiopia, Iraq, Nigeria, Pakistan, Somalia, and Syria, one delivered vaccines to adults at risk only). Half of the centres provided all vaccinations included in the NIP appropriate for age, while others offered only some vaccinations (Table 2). Informed consent was always required, written (19) or verbal (11). Some health services also reported that the staff could conduct outreach vaccination activities in migrant centres (five for migrant children and eight for migrant adults), especially if the number of migrants that needed to be vaccinated was high.

Data on administered vaccines were recorded by 35 out of 36 health services, and the information was made available by 30 health services (Table 5). Practical challenges faced by health services are listed in Table 3.

## 4. Discussion

As stated by WHO-UNHCR-UNICEF, vaccination access is recommended as part of the health support offered to migrants and should be performed with a systematic, sustainable and non-stigmatizing approach [12]. To our knowledge, this is the first study conducted in Italy to explore national and regional immunization policies targeting migrants and to assess how policies are implemented at local level.

We found that while in case of children the vaccination offer is widespread across Italy, different immunization policies are in place in the Italian Regions for adults. Vaccination against poliomyelitis and tetanus is offered to adults in all the responding Regions as recommended at national level, but only some of them offer MMR vaccination, which is considered a priority by WHO and ECDC, together with vaccination against polio. Some Regions offer more vaccinations than those recommended at the national level, some even offer all vaccinations included in the NIP appropriated for age. In addition, most of the community health services offer a range of vaccinations wider than what is provided by the national policies. The overprovision of vaccinations at regional and local levels compared to the national policies may be explained by several reasons: (i) the attempt to favour the integration of migrants in the community, by offering them all the vaccinations offered to the native population; (ii) the fear that migrants coming from highly endemic countries and living in overcrowded settings are at a higher risk of epidemic outbreaks; (iii) the fear of health threats brought by the mass immigration as the spreading of communicable diseases to the general population whose vaccination coverage is sub-optimal [32]. The observed overprovision is in line with European indications. In fact, although MMR and polio vaccines are a priority [12], a recent review on the current scientific evidence on vaccination in migrants and refugees states that, along with these vaccines, priority should be given also to HBV, diphtheria, tetanus and pertussis [33]. Furthermore, in densely populated settings, vaccinations against meningococcal disease, varicella, pneumococcus, and influenza during the cold season are also recommended [34,35]. Recently, the Italian guidelines for health checks and protection pathways for migrants on arrival have been published [36]. Recommendations concerning vaccination practices include all vaccinations in the NIP appropriate for age for unvaccinated children or children with an undocumented status (0–14 years), while, for adults, polio, diphtheria-tetanus-pertussis, MMR and varicella (except pregnant women), and HBV (after laboratory test of immunity) are indicated [36].

Asylum seekers, refugees, irregular migrants and unaccompanied minors can be moved from one reception centre to another, also across different Regions, and once obtained the asylum seeking or refugee status, they are free to move within the country [37]. Therefore, the regional heterogeneity we observed could impact the type of vaccination offer that adult migrants receive. Sharing of common indications on vaccinations offered to migrants should be encouraged.

Stating current recommendations, the vaccination offer provided according to the national legal framework should be updated, and include vaccines that are already offered by most (but not all) Regions, taking in consideration the availability of human and economic resources. The publication of the Italian guidelines [36] could set the direction of the national regulation.

We found that, in the whole Italian territory, vaccinations are delivered at the community level, mainly through public vaccination services. This is in line with international and Italian recommendations [12,36], and with policies in most European and Mediterranean countries [19,20] that do not recommend immunization at border crossings unless there is an outbreak. In fact, considering that intervals of months can be required between doses, the follow up of immunization series would be hard if the cycle has started at the entry point [12].

Few of the participating Regions/AA.PP and migrant centres reported to have developed procedures to facilitate the access of migrants to the community immunization services, such as dedicated staff to favour the access and fix a date for vaccination, or a systematic flow of information about migrants that need to be vaccinated. It represents a crucial point because it is known that many migrants have limited access to healthcare services because of legal, linguistic, and cultural barriers [13,19]. The local implementation of migrant-friendly procedures to guarantee migrant immunization should be supported.

International guidelines [12] indicate that tracking immunization data of migrant populations and exchange data on administered vaccines would allow appropriately planning immunization series and avoiding duplication of vaccination. We found that mechanisms to record and transmit data on administered vaccines are highly heterogeneous across Regions and local centres. A fundamental instrument to improve the homogeneity and coordination of the vaccination offer in Italy would be a national immunization electronic registry for recording vaccination of immunized migrants. This is a longstanding problem in Italy, where there is no national immunization electronic registry, even for Italian citizens [38]. The new Italian NIP 2017–2019 identified it as a critical issue that should be quickly implemented [39].

Furthermore, considering that Italy is part of the Schengen area, diversity in vaccination offer and data recording makes it more complicated to track migrants across countries to guarantee that migrants complete their immunization schedules and avoid re-vaccination [40]. Synergies in vaccination offer and appropriate mechanisms to promote collaboration and sharing of good practices are therefore needed foremost at the national level but also among different countries [40].

Recurring practical challenges in delivering the vaccination offer were consistently identified at all levels. Along with the scarce collaboration within health institutions and lack of operating procedures that have been highlighted by the fragmentation of the vaccination offer, other problems were low resources and need of specific training of HCW on migrant health. At the local level, some problems were reported that were not indicated at the national–regional level: in particular, low compliance of migrants to vaccination, logistic aspects, language barriers, and very scarce availability of prior immunization individual cards. Similar challenges were identified at the European level and the provision of interpreters, cultural mediators and information in the languages of the migrants were suggested as useful interventions to address the existing barriers to vaccination delivery and utilization [40]. Investigating real world challenges at the local level is essential for addressing the more pressing issues, and to allocate human and financial resources to improve migrants’ immunization offers.

Our survey presents several limitations. Firstly, it is not fully representative of the Italian situation. The absence of seven Regions may not give a complete picture of what happens in the Italian territory. Despite this, the responding Regions were well distributed in the Italian territory, covering areas of Northern, Central and Southern Italy and hosting 75% of the migrants arrived in Italy by the end of 2016 [2]. Health and migrant centres are also not representative. Even if contact points were identified in each Region, and all the governmental centres were contacted, participation in the survey was voluntary, and therefore some Regions are more represented than others. Furthermore, we could not stratify answers given at the local level by Region, given the small number.

## 5. Conclusions

This study provides an overview of immunization policies and practices targeting asylum seekers, refugees, irregular migrants and unaccompanied minors in Italy. The immunization provision for migrant children is guaranteed by national policies and is widespread at the local level. The national offer targeting adults is limited, but it is often increased by regional policies, although diversified.

Further efforts to ensure consistency in vaccine provision across Regions and local centres are needed to guarantee a complete vaccination offer and avoid unnecessary health actions, including unnecessary re-vaccination.

Fragmentation in data collection and recording has been documented at all levels. A national immunization electronic registry should be encouraged to record each vaccination administered, share data on vaccination coverage and monitor the immunization state.

Furthermore, difficulties that have been reported at the local level are not known at the central level. This gap should be bridged as these problems need to be addressed when considering national policies and resource allocations.

## Figures and Tables

**Table 1:** National and regional policies of immunization offer targeting migrants in Italy.

Question	National Level	N of Regions *	% **	Regions
Is there a regional regulation supporting immunization of migrants?				
None	-	6/14	43	LO, TN, TO, MA, CL, SA
Regional regulation specific for migrants ^	-	8/14	57	PI (2012) ^1^, BZ (2016), VE (2014), FVG (2016), ER (2014), UM (2015), PU (2009) SI (2011)
**CHILDREN/ADOLESCENTS**				
**Which are the target groups for vaccination?**				
**Immigration status**				
All (asylum seekers, refugees, irregular migrants, unaccompanied minors)	X	14/14	100	PI, LO, BZ, TN, VE, FVG, ER, TO, MA, UM, PU, CL, SI, SA
**Age group (years)**				
0–15		6/14	43	BZ, VE, UM, CL, SI, SA
0–18	X	8/14	57	PI, LO, TN, FVG, ER, TO, MA, PU
**Risk conditions**				
None	X	14/14	100	PI, LO, BZ, TN, VE, FVG, ER, TO, MA, UM, PU, CL, SI, SA
**Is the immunization status verified through anamnesis or check of vaccination card?**				
Yes	X	14/14	100	PI, LO, BZ, TN, VE, FVG, ER, TO, MA, UM, PU, CL, SI, SA
**Use of laboratory test if migrant has no immunization card?**				
Yes	X (HBV, tetanus)	9/13	69	PI (tetanus, measles, HBV), LO (tetanus, diphtheria), BZ (tetanus, diphtheria, HBV), TN (tetanus), FVG (tetanus, HBV, varicella), ER (tetanus, measles, rubella, HBV), UM ^2^, PU (IPV, MMR, varicella, HBV), SI ^2^
**Which vaccinations are offered to susceptible migrants?**				
All vaccinations included in the NIP	X	13/14	93	PI, LO, BZ, VE, FVG, ER, TO, MA, UM, PU, CL, SI, SA
IPV, tetanus, diphtheria, MMR		1/14	7	TN
**ADULTS**				
**Which are the target groups for vaccination?**				
**Immigration status**				
All (asylum seekers, refugees, irregular migrants)	X	14/14	100	PI, LO, BZ, TN, VE, FVG, ER, TO, MA, UM, PU, CL, SI, SA
**Age group (years)**				
No limits other than those in the NIP	X	14/14	100	PI, LO, BZ, TN, VE, FVG, ER, TO, MA, UM, PU, CL, SI, SA
**Risk conditions**				
None	X ^3^	13/14	93	PI, LO, BZ, TN, VE, FVG, ER, TO, MA, UM, PU, CL, SI
from polio endemic countries/countries at risk of polio reintroduction		1/14	7	SA
**Is the immunization status verified through anamnesis or check of vaccination card in ADULTS?**				
Yes	X (polio)	14/14	100	PI, LO, BZ, TN, VE, FVG, ER, TO, MA, UM, PU, CL, SI, SA
**Use of laboratory test if migrant has no immunization card?**				
Yes	X (HBV, tetanus)	4/14		BZ (tetanus, HBV), TN (tetanus), ER (tetanus, HBV, measles, rubella), CL (all included in the NIP, according to risks)
**Informed consent before vaccinating (both for ADULTS and CHILDREN)?**				
Yes, oral	n.a. ^4^	5/11	45	PI, TN, VE, FVG, MA
Yes, written		6/11	55	LO, BZ, ER, TO, PU, CL
**Sites for vaccination delivery (both for ADULTS and CHILDREN)?**				
Holding level	X	6/14	43	TN, VE, FVG, TO, PU, SI
Vaccination services	X	13/14	93	PI, LO, BZ, TN, VE, FVG, ER, TO, MA, UM, CL, SI, SA
Primary health care (GP/Paediatricians)		3/14	21	TO, PU ^5^, CL

* In some cases the denominator is not 14, representing the number of regions responding to each question; ** In some cases column percentages do not add up to 100% because answers to some questions were not mutually exclusive; ^ the year of publication of the regional regulation is reported among brackets; ^1^ A regional regulation has been released after the closure of the survey; ^2^ UM and SI stated that they use laboratory test but did not specify for which VPDs; ^3^ Particular attention paid to migrants from polio endemic countries or countries at risk for polio reintroduction, and those with wounds at risk for tetanus; ^4^ Not available (n.a.): as the variability of procedures at the regional and local level, the Italian responder could not provide this information; ^5^ Influenza and PCV vaccinations for adults are administered by GPs. Abbreviations: present (X) Lombardia (LO), Trento A.P. (TN), Toscana (TO), Marche (MA), Calabria (CL), Sardegna (SA), Piemonte (PI), Bolzano A.P. (BZ), Veneto (VE), Friuli Venezia Giulia (FNG), Emilia Romagna (ER), Umbria (UM), Puglia (PU), Sicilia (SI), National Immunization Plan (NIP), poliomyelitis (IPV), measles-mumps-rubella (MMR), hepatitis B (HBV).

**Table 2:** Vaccination offer to adult migrants documented as susceptible or with an undocumented immunization status, Italy, regions and at local level.

Vaccine	ITALY	REGIONS														LOCAL LEVEL	
		PI	LO	BZ	TN	VE	FVG	ER	TO	MA	UM	PU	CL	SA	SI	Migrant Centres (3) ^a^	Health Services (30) ^a^
Polio	**X**	X	X	X	X	X	X	X	X	X	X	X	X	X	X		14
Tetanus	**X ^b^**	X	X	X	X	X	X	X	X	X	X	X	X	X	X ^b^	2 *	28
Diphtheria		X	X	X	X	X	X	X	X	X	X	X	X			2 *	27
Pertussis							X	X	X	X		X	X			2 *	19
MMR	**X**			X		X	X	X	X	X	X	X	X			2 *	22
Varicella				X				X ^c^								2 *	18
Hepatitis B							X	X	X *	X *		X *	X *			2 *	20 *^,d^
Hepatitis A								X *	X *	X *		X *	X *			2 *	16 *
BCG ^e^																	
Influenza								X *	X *	X *		X *	X *			3 *	20 *
PCV				X				X *	X *	X *		X *	X *			2 *	16 *
Meningococcus C/ACWY								X *	X *	X *		X *	X *			2 *	15 *

^a^ the number indicates the number of migrant centres and health services; ^b^ in case of exposed wounds; ^c^ To women in fertile age; ^d^ three centres specified that is performed only to people negative to immunization check through laboratory testing; ^e^ Bacillus Calmette-Guerin; * Four Regions, one migrant centre and 15 health services, limited this offer to population at-risk according to NIP. Abbreviations: present (X).

**Table 3:** Practical challenges in migrants’ vaccination at national, regional and local level.

Challenges	ITALY	REGIONS	LOCAL LEVEL	
			Migrant Centres (28) ^a^	Health Services (36) ^a^
Scarcity of resources	**X**	PI, LO, TN, ER, TO, PU, CL, SA	6	17
Need of specific training of health care workers on migrant health	**X**	LO, TO, MA, SA	3	11
Lack of operating procedures	**X**	PI, MA, CL	4	8
Scarce collaboration within health institutions		PI, LO, MA, TO	7	5
Low compliance of migrants to vaccination		VE	5	7
Lack of health staff		BZ		
Logistic issues			4	2
Waiting time			1	
Difficulties due to the short time of staying of migrants and the frequency of relocation			1	1
Language barriers				3

**^a^** the number indicates the number of migrant centres and health services. Abbreviations: present (X).

**Table 4:** Characteristics of responding migrant reception centres and community health services.

Total No. of Responding Centres		Migrant Centres (28) ^a^	Health Services (36) ^a^	
Dealing with children/adolescents		15 ^b^	35	
Dealing with adults		25	32	
Performing Health assessment		23	23	
Giving a personal health card to migrants		23	22	
Available cultural mediators		28	13 ^c^	
Length of stay of migrants in the centre ^d^	<6 months	6		
	6–12 months	12		
	>12 months	10		
Maximum capacity of the centre ^d^	<50 people	9		
	51–150	11		
	151–300	1		
	>300	7		
Presence of an outpatient clinics inside the centre ^d^		22		
Who provides health services? ^d^	PHC services	22		
	NGOs	7		
	Staff of the centre	5		

^a^ the number indicates the number of migrant centres and health services; ^b^ all Hubs were hosting minors; CIE wasn’t; ^c^ four other centres explained that the staff of the migrant centres helped in translating; ^d^ these questions were addressed to migrant centres only.

**Table 5:** Recording and transmission of information on administered vaccines, migrant reception centre and community health services.

		Migrant Reception Centres (5) *	Community Health Services (36) *
**Is information on vaccine administration recorded?**			
**Yes (specify where, more than one answer possible)**		**5**	**35 ^1^**
	Individual health record delivered to migrants	4	18
	Electronic archive dedicated to migrants	2	9
	Paper archives dedicated to migrants	3	8
	General population electronic immunization registries	3	25
	General population paper-based immunization registries	2	4
**Is information on vaccine administration made available to other institutions?**			
**Yes (specify to whom, more than one answer possible)**		**5**	**30 ^1^**
	It follows the same flow of the information on immunization of general population	-	18
	To migrants’ holding centres	-	15
	To LHU vaccination services	4	9
	To GPs and paediatricians	-	5
	To holding centres where migrants are relocated	4	6
	To local health authorities	1	3
	To regional health authorities	-	7
	To international institutions	1 (IOM)	-

* only migrant centres and community health services administering vaccines; ^1^ one health service did not answer to these questions.
